# Platelet Content of Nitric Oxide Synthase 3 Phosphorylated At Serine^1177^ Is Associated with the Functional Response of Platelets to Aspirin

**DOI:** 10.1371/journal.pone.0082574

**Published:** 2013-12-20

**Authors:** Javier Modrego, Luis Azcona, Naiara Martín-Palacios, José J. Zamorano-León, Antonio Segura, Pablo Rodríguez, Reddy Guerra, Juan Tamargo, Carlos Macaya, Antonio J. López-Farré

**Affiliations:** 1 Cardiovascular Research Unit, Hospital Clínico San Carlos, Universidad Complutense de Madrid, Madrid, Spain; 2 Hemodynamic Unit, Cardiology Department, Hospital Clínico San Carlos, Universidad Complutense de Madrid, Madrid, Spain; 3 Health Science Institute, Talavera de la Reina, Toledo, Spain; 4 Pharmacology Department, School of Medicine, Instituto de Investigación Sanitaria del Hospital Clínico San Carlos (IdISSC), Universidad Complutense de Madrid, Madrid, Spain; University of Alabama at Birmingham, United States of America

## Abstract

**Objective:**

To analyse if platelet responsiveness to aspirin (ASA) may be associated with a different ability of platelets to generate nitric oxide (NO).

**Patients/Methods:**

Platelets were obtained from 50 patients with stable coronary ischemia and were divided into ASA-sensitive (n = 26) and ASA-resistant (n = 24) using a platelet functionality test (PFA-100).

**Results:**

ASA-sensitive platelets tended to release more NO (determined as nitrite + nitrate) than ASA-resistant platelets but it did not reach statistical significance. Protein expression of nitric oxide synthase 3 (NOS3) was higher in ASA-sensitive than in ASA-resistant platelets but there were no differences in the platelet expression of nitric oxide synthase 2 (NOS2) isoform. The highest NOS3 expression in ASA-sensitive platelets was independent of the presence of T-to-C mutation at nucleotide position −786 (T^−786^→C) in the NOS3-coding gene. However, platelet content of phosphorylated NOS3 at Serine (Ser)^1177^, an active form of NOS3, was higher in ASA-sensitive than in ASA-resistant platelets. The level of platelet NOS3 Ser^1177^ phosphorylation was positively associated with the closure time in the PFA-100 test. *In vitro*, collagen failed to stimulate the aggregation of ASA-sensitive platelets, determined by lumiaggregometry, and it was associated with a significant increase (p = 0.018) of NOS3 phosphorylation at Ser^1177^. On the contrary, collagen stimulated the aggregation of ASA-resistant platelets but did not significantly modify the platelet content of phosphorylated NOS3 Ser^1177^. During collagen stimulation the release of NO from ASA-sensitive platelets was significantly enhanced but it was not modified in ASA-resistant platelets.

**Conclusions:**

Functional platelet responsiveness to ASA was associated with the platelet content of phosphorylated NOS3 at Ser^1177^.

## Introduction

Nitric oxide (NO) is generated by conversion of L-arginine into L-citrulline and, in platelets, it seems to be predominantly produced by the activity of a constitutive NO synthase 3 (NOS3), although small amounts of an inducible NOS (NOS2) isoform have also been detected [1], [2]. Platelet-derived NO acts as negative feedback mechanisms reducing the recruitment of new platelets to the growing thrombus [3], [4]. Indeed, it has been suggested that alterations in NO biosynthesis by platelets may contribute to enhance thrombotic processes and coronary events [5].

Different factors may influence NOS3 activity. Among them, endogenous circulating L-arginine competitive antagonists, such as asymmetric dimethylarginine (ADMA), have been reported increased in pathophysiological conditions in which NO production was reduced [6]. Moreover, in human platelets, ADMA may also inhibit L-arginine transport [7].

The different expression level of NOS3 protein may also influence the ability of platelets to produce NO. In this regard, there is evidence that the expression level of NOS3 protein could be modulated by several factors, including genetic factors. Indeed, the T-to-C mutation at nucleotide position −786 (T^−786^→C) in the 5′-flanking region of NOS3-coding gene, reduced the promoter activity of NOS3 gene, compromising NOS3 expression and, therefore, NO synthesis [8].

The NOS3 activity regulation, it is not only important to protect NOS3 expression but also to induce NOS3 phosphorylation. Interestingly, it has been demonstrated that stimulation of NOS3 activity is tightly dependent of its phosphorylation state and even with greater extent than for other NOS isoforms [9]. In this regard, different amino acid residues may be phosphorylated on NOS3 sequence but, in humans, phosphorylation of NOS3 protein at Serine (Ser)^1177^, promotes NOS3 activity [10].

Aspirin (ASA) is an effective antiplatelet drug that inhibits platelet thromboxane A_2_ (TXA_2_) production. However, despite of the clinical benefits of ASA, a number of ASA-treated patients showed insufficient inhibition of their platelets. Indeed, a close relationship between platelet resistance to ASA and increased incidence of cardiovascular events has been established [11].

Several mechanisms have been postulated to explain these reduced platelet response to ASA. Among them, non-treatment compliance, genetic-related alterations associated with TXA_2_ production, a different platelet energetic metabolism and an increased platelet turnover have been reported [12]–[14].

Previous studies have demonstrated that ASA also stimulates NOS3 activity in platelets increasing platelet NO production and favouring the antiplatelet effects of ASA [15]. However, in our knowledge, it has not been analysed if NO produced from platelets may be associated with the platelet responsiveness to ASA. Therefore, the aim of the present study was to analyze whether the different response of platelets to ASA may be associated with a different ability of platelets to produce NO.

## Materials and Methods

### Patients

Caucasian clinically stable coronary ischemic patients (n = 50) taking ASA were consecutively included in the study. Patients were divided as ASA-resistant (n = 24) and ASA-sensitive (n = 26) based on platelet functionality according to the PFA-100 test.

All the included patients had been taking 100 mg/day ASA from at least the last nine months and the coronary acute event had been occurred at least nine months before inclusion. The occurrence of an acute cardiovascular event during the nine months before inclusion was considered as exclusion criteria. Patients were also excluded if they were under other antithrombotic drugs treatment or non-steroid anti-inflammatory drugs within 30 days before inclusion and if they were under nitrate therapy. Exclusion criteria were also patients showing thrombocytopenia, anemia or plasma creatine ≥2 mg/dL.

Blood samples were collected in tubes containing 10% v/v acid-citrate dextrose by antecubital venepuncture, in the morning 2 to 4 hours after the last ASA dose was taken. The initial 3 to 4 mL of blood was discarded to reduce spontaneous platelet activation. The study was performed conformed to the Declaration of Helsinki and the institutional review board of Hospital Clinico San Carlos approved this study. All patients gave signed consent.

### Identification of ASA resistance platelets

As above mentioned, ASA-resistant patients were identified by using the PFA-100 assay (Dade Behring W. Sacramento, USA). This test has been used to predict clinical recurrences in cardiovascular patients under ASA treatment [16].

As we previously reported [12], [17], for PFA-100 assays disposable cartridges containing collagen-coated membrane infused with epinephrine (10 μg) were used. The time to occlude a small pore (150 μm) contained in a membrane localized into the cartridges when whole blood was infused into it was defined as closure time (CT). Therefore, CT is indicative of platelet function for the whole blood sample. According to manufacturer, CT ranges from 94 to 193 seconds with epinephrine-cartridges defined ASA-resistant platelets. In ASA-sensitive platelets, epinephrine-CT is prolonged. After 300 seconds, the processes automatically terminate.

As we previously reported [12], [17], to discard the effect of non-compliance in the lack of response of platelets to ASA, PFA-100 assay was performed at inclusion and one hour after patients received an additional ASA dose (100 mg). Only patients that demonstrated similar CT range at inclusion and one hour after the additional 100 mg ASA administration were considered for the study.

### Platelet isolation and purity

Whole blood samples were centrifugated at 1000 rpm for 15 minutes at 20°C. Platelet-rich plasma (PRP) was then recovered and divided into aliquots containing 1.25×10^8^ platelets. The final volume was adjusted to 500 µL with RPMI-1640 without red phenol. Platelets were then incubated at 37°C for 20 minutes with continuous stirring (1000 rpm). After incubation, PRPs were centrifugated at 2500 rpm at 4°C and the supernatants and the platelet pellets were separately recovered and frozen at −80°C until the biochemical and molecular biology determinations were performed.

The purity of platelets in the PRP samples was further evaluated by flow cytometry assays. For this purpose, PRP was incubated with fluorecein isothyocyanate-conjugated monoclonal antibodies against CD61 (130–081–501, Miltenyi Biotec, Auburn, CA, USA), CD45 (5450-PE100T, BioCytex, Marseille, France) and CD235-A (340947, Becton-Dickinson, San Jose, CA, USA) that respectively identified platelets, leukocytes and erythrocytes. The samples were then analyzed in a flow cytometer (FACSCalibur, Becton-Dickinson, San Jose, CA, USA).

### Nitrite + nitrate released from platelets

In the platelet supernatants, nitrite + nitrate were measured using a colorimetric assay kit (78001, Cayman Chemical Company, Ann Arbor, MI, USA) following manufactureŕs instructions. The sensitivity of the assay was 2,5 μmol/L. The intra and inter- assay variations coefficients were 2.7 and 3.7% respectively.

### Determination of NOS2, NOS3 and phosphorylated NOS3 at Ser^1177^ by Western blotting

The amount of NOS2, NOS3 and phosphorylation at Ser^1177^ residue in NOS3 were analysed by Western blot. For this purpose, 20 μg of total platelet proteins, estimated by bicinchoninic acid reagent (Pierce), were developed in 10% SDS/PAGE [18]. Parallel gels to determine β-actin were also developed for control protein loading. The gels were then blotted onto nitrocellulose membranes. Membranes were then overnight blocked with 5% (w/v) bovine serum albumin and incubated with monoclonal antibodies against NOS3 (sc-653, Santa Cruz Biotechnology, Inc. Santa Cruz, CA, USA, dilution 1∶500), NOS2 (sc-8310, Santa Cruz Biotechnology, USA, dilution 1∶1500), anti-phosphorylated form NOS3-Ser^1177^ (804–396C100 Alexis Biochemical, California USA, dilution 1∶1000) and anti-β-actin (A-5441, Sigma-Aldrich, St. Louis, MO, USA, dilution 1∶1000) respectively. Nitrocellulose membranes were then revealed with peroxidase-conjugated anti-rabbit IgG (1∶2500 for NOS3 and NOS2) and peroxidase-conjugated anti-mouse IgG (1∶1500 for NOS3 Ser^1177^ and 1∶7500 for β-actin). The signal of the protein expression level was revealed using enhanced chemiluminiscence reagents (ECL; GE Healthcare, Little Chalfont Buckinghamshire, UK) and detected in a transilluminator (Gel Logic 440 imagin system, Kodak, USA).

### Determination of NOS3 gene T^−786^→C

Genomic DNA was extracted from subjectś white blood cells as previously reported [19]. The 5′-flaking region of NOS3 was amplified by polymerase chain reaction (PCR). The sequence of the primers used was: forward: 5′-GTGTACCCCACCTGCATTCT-3′; reversal: 5′- GGGACACAAAAGAGCAGGAA-3. PCR conditions were initial denaturation at 95°C for 5 min followed by 40 cycles of denaturation for 30 s at 95°C, anneling for 1 min at 52°C and extension for 1 min at 72°C. The PCR products were then purified using a commercial kit (Ultra Clean PCR Clean-up DNA purification kit, Carlsbad, CA) and sequenced with Big-Dye terminator chemistry in both directions using an ABI PRISM 310 Automatic DNA sequencer (Applied Biosystems, Foster City, CA).

### Determination of ADMA levels in plasma

ADMA plasma levels were determined using a commercial ELISA kit (Diagnostika GMBH, Hamburg Germany) following the manufactureŕs recommendations. The sensitivity of the assay was 0.05 μmol/L. The inter- and intra- assay variation coefficients were 9,4% and 6.0% respectively.

### Platelet aggregation in response to collagen

PRP obtained from an additional subgroup of consecutively included ASA-sensitive (n = 10) and ASA-resistant (n = 10) patients was undergoing to *in vitro* aggregation induced by collagen, an ASA-inhibitable inductor of platelet aggregation. Platelet aggregation was recorded using a lumiaggregometer (Aggrecorder, two channels). Platelet-poor plasma (PPP) was used as control for 100% light transmission. PRP containing 1,25×10^8^ platelets was adjusted to 600 µL with PPP and incubated in the aggregometer at 37°C for 20 min with continuous stirring (1000 rpm). After this time, an aliquot (100 µL) was removed to determine nitrite + nitrate concentration. This aliquot was centrifugated (2500 rpm, 10 min at 4°C) and the pellet and supernatant separately frozen at −80°C.

The reminder 500 µL PRP was then stimulated with submaximal collagen concentrations (0.5, 1.5 and 3.5 µg/mL) in accumulative form for 20 min. These collagen concentrations were chosen based on a previous reported observation showing that induced different degree of change on light transmission between ASA-responder and ASA non-responders platelets [20]. After collagen incubation, the PRP was recovered, centrifuged (2500 rpm for 10 min at 4°C) and the supernatant and pellet frozen separately at −80°C for determination of nitrite + nitrate and NOS3 phosphorylated at Ser^1177^ respectively.

### Statistical analysis

Values are expressed as mean ± (standard error of the mean) S.E.M. Mann-Whitney test was used to compare the continuous variables between the two experimental groups. The adjusted association between the biochemical parameters with the platelet response to ASA was analysed by using a conditional logistic-regression model with ASA resistance as dependent variable, the biochemical parameters as independent variable and angiotensin I-converting enzyme inhibitors (ACEI) treatment as covariate. Correlations were performed using Pearsońs analysis. The statistical analysis was performed using SPSS 15.0. A p value <0.05 was considered statistically significant.

## Results

Clinical features of the patients with ASA-resistant and ASA-sensitive platelets are shown in table 1. Only patients with extreme CT values for each of the ASA responsiveness conditions were included in the study (Table 1).

**Table 1:** Clinical features and pharmacological treatment of patients with ASA-sensitive and ASA-resistant platelets.

Parameters	ASA-sensitive(n = 26)	ASA-resistant(n = 24)
**Age (years)**	65.4±1.9	68.7±1.5
**Gender (Male/female)**	26/0	22/2
**CT values (s)**	>300	124.8±7.1
**Hypertension n (%)**	16 (61.5)	13 (54.1)
**Hyperlipemia n (%)**	15 (57.7)	14 (58.3)
**Diabetes mellitus n (%)**	6 (23.1)	9 (37.5)
**Pharmacological treatments**		
Aspirin (%)	26 (100)	24 (100)
β-blockers (%)	18 (69.2)	18 (75.0)
ACE inhibitors n (%)	15 (57.7)	6 (25.0)*
Statins (%)	26 (100)	24 (100)
Diuretics (%)	7 (26.9)	5 (20.8)

ACE: angiotensin-converting enzyme. CT: closure time after one hour of the last ASA administration. CT is the time necessary for the occlusion of the open pore in the PFA-100 cartridges and it is indicative of platelet function. The upper limit of the PFA-100 test is 300 seconds. The results of categorical variables are represented as number of cases with respect to the total included patients within each experimental group. Age is represented as mean ± SEM. *p<0.05 with respect to patients with ASA-sensitive platelets.

The included patients with platelet resistant and sensitive to ASA showed similar clinical history of cardiovascular risk factors (Table 1). More patients with ASA-sensitive platelets were under ACEI treatment as compared with those with ASA-resistant platelets (Table 1). Other pharmacological treatments were similar between the two experimental groups (Table 1).

**Figure 1:**
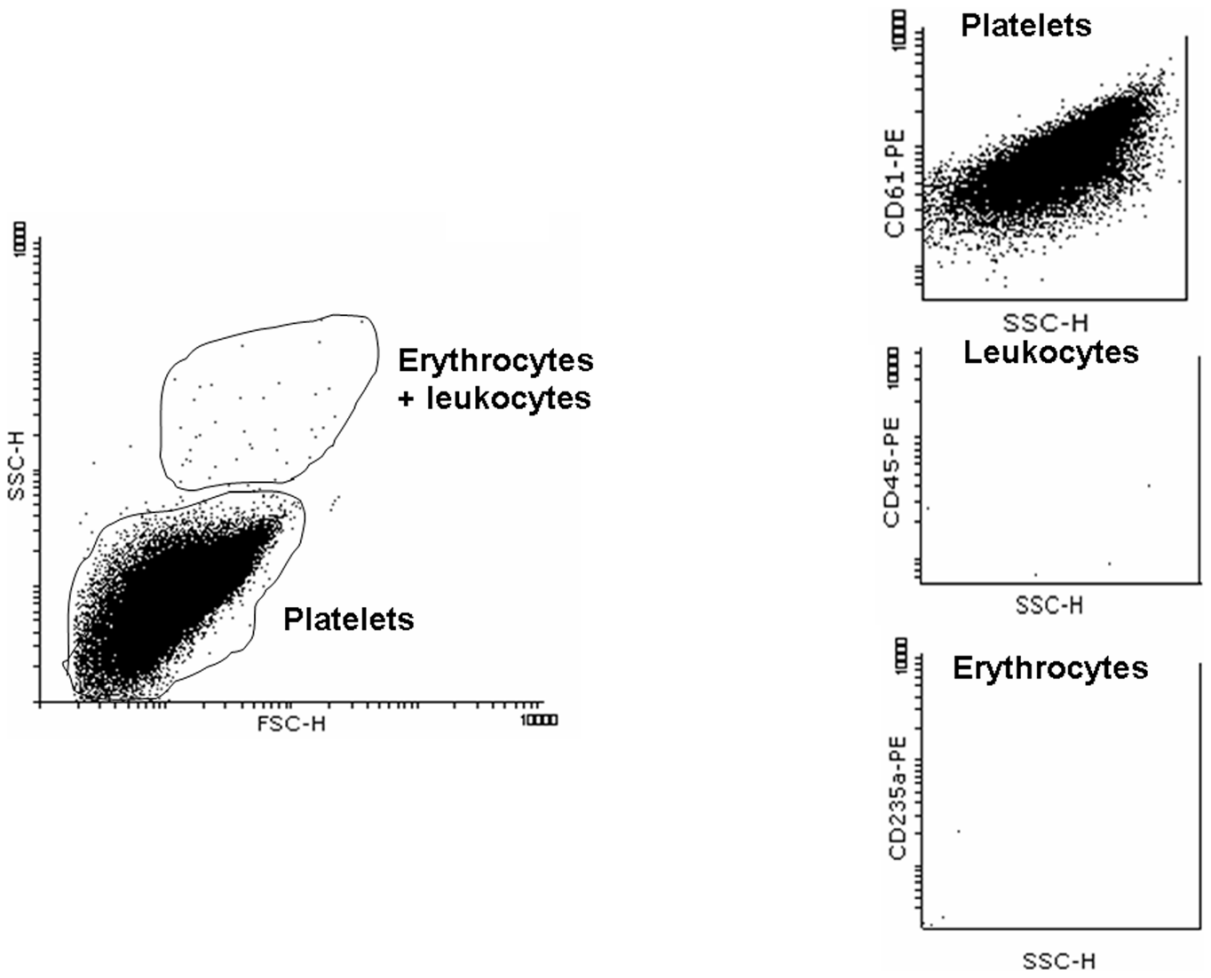
Flow cytometry analysis of platelets, leukocytes and erythrocytes in the PRP. On the left, representative flow cytometry graph to detect platelet population according to size-scatter and forward-scatter. On the right, representative flow cytometry graphs to detect positivity to CD61, CD45 and CD235a in the PRP that stain platelets, leukocytes and erythrocytes respectively.

The purity of the platelet samples was tested by flow cytometry. Figure 1 illustrates a representative flow cytometric analysis by the size and positively staining to CD61^+^ (platelet stained), CD45^+^ (leukocyte stained) and CD235-A^+^ (erythrocyte stained). Erythrocytes and leukocytes constituted less than 0,05% of the PRP sample and more of 99,7% was constituted by platelets.

### Nitrite + nitrate production and NOS3 and NOS2 expression in platelets

In supernatants from ASA-sensitive platelets, the detected amount of nitrite + nitrate tended to be higher than in ASA-resistant platelets but it did not reach statistical significance (nitrite + nitrate in μmol/L: ASA-sensitive: 15.49±2.65, ASA-resistant: 11.63±2.40; p = 0.09). This finding was not modified after adjustment by ACEI treatment (p = 0.315).

**Figure 2:**
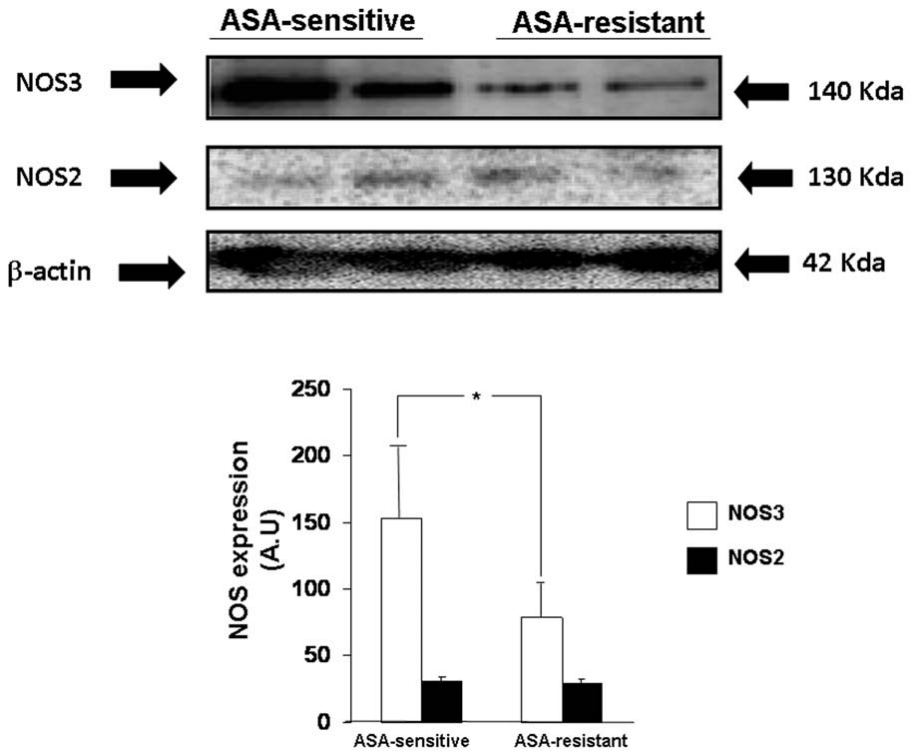
Representative Western blots showing the expression of NOS2 and NOS3 proteins in platelets obtained from two representative patients with ASA-sensitive and ASA-resistant platelets. The expression of β-actin was used as loading protein control. At the bottom, bar graphs showing the densitometric analysis of the all the Western blots represented in arbitrary units (A.U.). Results are represented as mean ± SEM *p<0.05 with respect to ASA-sensitive platelets.

A significant higher expression of NOS3 protein was found in ASA-sensitive than in ASA-resistant platelets (Figure 2). The highest NOS3 expression in ASA-sensitive platelets was independent of ACEI treatment since NOS3 expression remained higher in ASA-sensitive than in ASA-resistant platelets when in the logistic-regression model NOS3 expression was adjusted for ACEI treatment (p = 0.049).

Pearsońs analysis demonstrated a negative correlation between the expression levels of NOS3 protein in platelets and CT values in the PFA-100 assay (Pearsońs coefficient =  −0.3; p = 0.048).

In the platelets, the level of expression of NOS2 protein was weak and there were no differences between ASA-sensitive and ASA-resistant platelets (Figure 2).

### NOS3 gene T^−786^→C mutation

Nucleotide sequencing of NOS3 gene at −786 position revealed that most of the patients showed homozygosis for CC and there were no differences between patients with ASA-sensitive and ASA-resistant platelets (Table 2). Moreover, similar number of ASA-sensitive and ASA-resistant patients showed TT and TC alleles at −786 in NOS3 gene (Table 2).

**Table 2:** Nucleotide sequence at −786 position in the 5′-flaking region of NOS3 gene.

	NOS3 gene, −780 T>C			
	CC	TC	TT	Total
***ASA-sensitive platelets (n = 26)***	14	7	5	26
***ASA-resistant platelets (n = 24)***	14	7	3	24

### Platelet content of phosphorylated NOS3 at Ser^1177^

**Figure 3:**
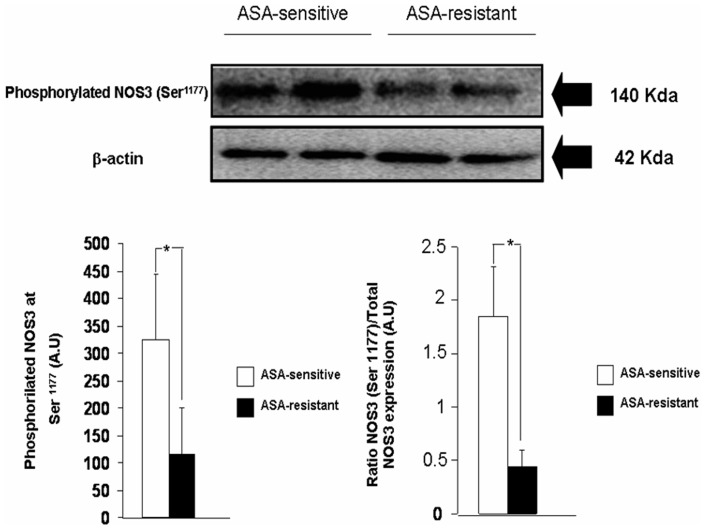
Representative Western blots to determine the platelet content of phosphorylated NOS3 at Ser^1177^ in ASA-sensitive and ASA-resistant platelets. The expression of β-actin was used as loading protein control. At the bottom, on the left the bar graphs shown the densitometric analysis of the all the Western blots. On the right the densitometric analysis of platelet content of phosphorylated NOS3 Ser^1177^ in ASA-sensitive and ASA-resistant platelets was ccalculated as ratio of total NOS3 expressed in ASA-sensitive and ASA-resistant platelets. The densitometric analyses are shown as densitometric arbitrary units (A.U.). Results are represented as mean ± SEM *p<0.05 with respect to ASA-sensitive platelets.

Platelet content of phosphorylated NOS3 at Ser^1177^ was significantly higher in ASA-sensitive than in ASA-resistant platelets (Figure 3). It was also evident when the platelet content of phosphorylated NOS3 Ser^1177^ was calculated as ratio related to the respective total NOS3 expressed in ASA-sensitive and ASA-resistant platelets (Figure 3). After adjustment by ACEI treatment, the level of NOS3 Ser^1177^ was also significantly higher in ASA-sensitive than in ASA-resistant platelets (p = 0.04).

Pearsońs analysis showed a positive correlation between CT values in PFA-100 assay and platelet content of NOS3 Ser^1177^ (Pearsońs coefficient: 0.546; p = 0.035).

### ADMA plasma levels

In plasma, ADMA levels were no different between patients with ASA-resistant and ASA-sensitive platelets (ADMA in μmol/L: ASA-sensitive: 0.28±0.02, ASA-resistant: 0.25±0.02; pNS). The lack of difference in ADMA plasma levels was also maintained after adjustment by ACEI treatment (p = 0,456). Moreover, Pearsońs analysis demonstrated non-association between ADMA plasma levels and CT values in the PFA-100 test (Pearsońs coefficient: 0.086; p = 0.557).

### Changes on light transmission, nitrite + nitrate release and phosphorylated NOS3 Ser^1177^ content in collagen-stimulated platelets

On the light of the observed results, it was speculated if phosphorylated NOS3 Ser^1177^ content may be different in conditions of platelet stimulation and if it was associated with the ability of ASA to inhibit platelet activity. Therefore, an additional new group of stable coronary ischemic patients taking aspirin (100 mg/day for at least the last nine months), who clinical features are showed in table 3, were recruited and their platelets classified by the PFA-100 test as ASA-sensitive (n = 10) and ASA-resistant (n = 10). PRPs obtained from both groups of patients were then *in vitro* stimulated with increasing collagen concentrations.

**Table 3:** Clinical features and pharmacological treatment of ASA-sensitive and ASA-resistant patients undergoing to in vitro stimulation to collagen.

Parameter	ASA-sensitive (n = 10)	ASA-resistant (n = 10)
**Age (years)**	66.1±2.8	70.0±1.9
**Gender (Male/female)**	10/0	10/0
**CT values (s)**	>300	109.3±12.5
**Hypertension n (%)**	6 (60.0)	4 (40.0)
**Hyperlipemia n (%)**	6 (60.0)	5 (50.0)
**Diabetes mellitus n (%)**	2 (20.0)	4 (40.0)
**Pharmacological treatment**		
Aspirin (%)	10 (100)	10 (100)
β-blockers (%)	7 (70.0)	9 (90.0)
ACE inhibitors n (%)	6 (60.0)	3 (30.0)
Statins (%)	10 (100)	10 (100)
Diuretics (%)	1 (10.0)	2 (20.0)

ACE: angiotensin-converting enzyme. CT: closure time. The results of categorical variables are represented as number of cases with respect to the total included patients within each experimental group. Age is represented as mean ± SEM.

In ASA-sensitive platelets, addition of increasing collagen concentrations (0.5 to 3.5 µg/mL) failed to modify light transmission, suggesting that the platelet aggregatory response to collagen was almost abolished (Table 4). However, in ASA-resistant platelets submaximal collagen concentrations reduced light transmission in PRP suggesting that ASA-resistant platelets were more sensitive to collagen than ASA-sensitive platelets (Table 4).

**Table 4:** Changes of light transmission and nitrite + nitrate released from collagen-stimulated platelets with collagen.

	% light transmission				Nitrite + nitrate released (µmol/L)	
	Baseline	0.5 μg/ mL Collagen	1.5 μg/ mL Collagen	3.5 μg/ mL Collagen	Baseline	3.5 μg/ mL Collagen
***ASA-sensitive (n = 10)***	0	0.86±0.50	0.57±0.32	0.86±0.60	13.01±3.40	23.38±2.18*
***ASA-resistant (n = 10)***	0	10.43±2.83*	10.86±2.80*	12.71±3.18*	11.76±2.51	12.13±1.59

Results are represented as mean ± SEM. * P<0.05 with respect to the corresponding baseline.

As above observed, in the absence of collagen (resting conditions), the amount of nitrite + nitrate released from ASA-sensitive was not different to that released from ASA-resistant platelets (Table 4). During collagen (3.5 µg/mL) stimulation ASA-sensitive platelets released significantly more nitrite + nitrate than that observed before collagen addition (Table 4). However, in ASA-resistant platelets collagen addition failed to modify the release of nitrite + nitrate with respect to that measured before collagen (Table 4).

**Figure 4:**
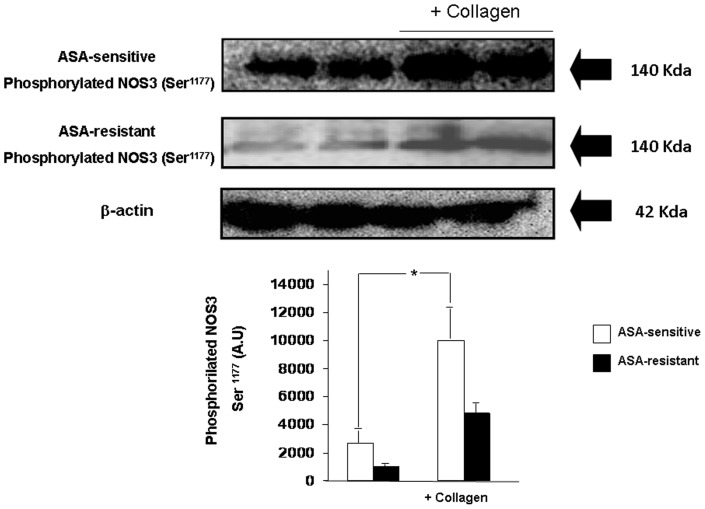
Representative Western blots to determine the platelet content of phosphorylated NOS3 at Ser^1177^ before and after collagen (3.5 µg/mL) stimulation of ASA-sensitive and ASA-resistant platelets. The expression of β-actin was used as loading protein control. At the bottom, is represented the densitometric analysis of all the Western blots (ASA-sensitive n = 10; ASA-resistant n = 10). The densitometric analysis is shown as densitometric arbitrary units (A.U.). Results are represented as mean ± SEM *p<0.05 with respect to the corresponding experiment in absence of collagen.

In ASA-sensitive platelets, collagen (3.5 µg/mL) significantly (p = 0.032) increased the platelet content of phosphorylated NOS3 Ser^1177^ as compared with that observed before collagen (Figure 4). However, as figure 4 shows in ASA-resistant platelets, collagen (3.5 µg/mL) only slightly increased the platelet content of phosphorylated NOS3 Ser^1177^ as compared with that observed before collagen stimulation and it did not reach statistical significance (p = 0,568).

## Discussion

The present work shows for the first time that ASA responder platelets contain higher amount of phosphorylated NOS3 at Ser^1177^ protein, an active form of human NOS3, than ASA-resistant platelets but under resting conditions did not produced higher amount of NO than ASA-resistant platelets. However, during *in vitro* stimulation of ASA-sensitive platelets with collagen both the release of NO and the platelet content of phosphorylated NOS3 Ser^1177^ was significantly higher (p = 0.018) than ASA-resistant platelets and it was associated with suppression of the aggregating response to collagen.

Several studies have demonstrated that ASA stimulates NO production in different cells including platelets [15], [21]. NO synthetized by platelets has an important role as regulator of platelet activation since NO inhibited platelet aggregation and platelet recruitment to growing thrombus [3], [4].

In the present study, despite of NOS3 expression level was higher in ASA-sensitive than in ASA-resistant platelets, the ability of ASA-sensitive platelets to produce NO, determined as nitrite + nitrate content in the platelet supernatants, was not significantly different with respect to ASA-resistant platelets. It suggests that in resting conditions, the ability to produce NO from ASA-sensitive platelets may be attenuated.

The first question raised from these results is why NO activity seems to be attenuated in ASA-sensitive as compared with ASA-resistant platelets. There are several factors involved on the regulation of NOS3 activity including ADMA, an endogenous inhibitor of NOS activity [6]. In platelets, ADMA not only antagonized the conversion of L-arginine into L-citrulline but also reduced L-arginine influx into them [7]. In the present study, similar plasma levels of ADMA were observed between patients with ASA-sensitive and ASA-resistant platelets. It diminishes the possibility that in PRP the production of NO in ASA-sensitive and ASA-resistant patients may be modulated by different ADMA levels.

### NOS3 phosphorylation is another important factor to regulate NOS3 activity

In this regard, it is well known that when NOS3 is phosphorylated at Ser^1177^ position, the flux of electrons through the reductase domain is increased and, consequently, the NO production [22]. Indeed, activation of NOS3 in platelets has been linked to phosphorylation of NOS3 at Ser^1177^. However, in our experiments, ASA-sensitive platelets showed higher content of phosphorylated NOS3 at Ser^1177^ than ASA-resistant platelets but their apparent ability to produce NO was not significantly different between them.

Although in platelets the importance of NOS3 phosphorylation on the regulation of NOS3 activity is well accepted, in our knowledge, phosphorylation at Ser^1177^ residue is the only currently established in human platelets [23]. This contrast with NOS3 in endothelial cells where other phosphorylation sites have been identified and even some of them associated with reduction of NOS3 activity [24]. Therefore, it may be plausible that in ASA-sensitive platelets other putative phosphorylation sites in NOS3 protein may counterbalance NOS3 Ser^1177^ phosphorylation and, therefore, attenuate NOS3 activity. In addition, since a complex variety of NOS3-interacting proteins and cofactors are involved in the regulation of NOS3 activity, with the present experiments we can not discard that any of them may be involved in the attenuation of platelet NOS3 activity in ASA-sensitive platelets. Further specific experimental works are needed to clarify it.

The second question raised from our findings is why NOS3 expression was higher in ASA-sensitive than in ASA-resistant platelets. In this regard, it has been demonstrated that T^−786^→C mutation in NOS3 gene promoter reduced NOS3 expression [8]. Most of half of the here studied stable coronary ischemic patients showed homozygosis for CC at −786 in the 5-flaking region of NOS3 gene which it is in accordance with previous works demonstrating that T^−786^→C mutation in NOS3 gene promoter is associated with coronary ischemia and coronary spam [8], [25]. However, alleles distribution at −786 site in NOS3 promoter did not reveal differences between patients with ASA-sensitive and ASA-resistant platelets, discarding this genetic alteration as contributor of the different expression of NOS3 protein between them.

In addition to genetic factors, there are other factors that may regulate NOS3 expression. Among them inflammatory-related factors have demonstrated to alter NOS3 expression [26]. Although it was observed that highest quartile of C-reactive protein elevation showed a significant benefit from aspirin treatment [27], it is not clear whether platelet resistance to ASA may be associated with systemic inflammation. In this regard, although some works have postulated a relationship between inflammation and platelet responsiveness to ASA others, including proteomic study in plasma reported by us, could not found it [17], [28], [29]. At this point, it is also interesting to remind that NO-related mechanisms may serves as negative-feedback regulator of NOS3 expression [30]. Therefore, as speculation, the attenuated ability to produce NO by ASA-sensitive platelets may favour by itself NOS3 expression in them.

With the present experimental design it could be not able to discard that modulation of NOS3 protein expression in platelets may be occurring in the mature platelets since platelets contain a number of mRNAs including NOS3 mRNA [1], [31]. However, it is also plausible that the different expression of NOS3 protein may be consequence of a different type of platelets produced at megakaryocyte level. Accordingly, we have recently reported that others constitutive platelet proteins, such as those related to energetic metabolism, oxidative stress and cell survival processes, have also a different level of expression in ASA-sensitive than in ASA-resistant platelets [13]. Further studies are needed and warranted to analyse the mechanisms that promote highest NOS3 expression in ASA-sensitive platelets.

As mentioned, our findings could seem difficult to explain, since although highest NOS3 Ser^1177^ phosphorylation content in platelets was positively associated with the functional platelet response to ASA, the ability to produce NO from ASA-sensitive platelets was not significantly different than that in ASA-resistant platelets. Therefore, we further analysed the possible significance of these findings during platelet activation.

Collagen is a known ASA-inhibitable stimulator of platelet activation but collagen also promotes platelet NO synthesis, probably as mechanisms to limit collagen-dependent platelet activation [32]. During collagen-stimulation of ASA sensitive platelets, NOS3 Ser^1177^ phosphorylation was enhanced with respect to that found in these platelets at resting situation. However, during collagen stimulation of ASA-resistant platelets only a slight increase of NOS phosphorylation at Ser^1177^ was observed. Furthermore, in response to collagen, ASA-resistant platelets did not produce further amount of nitrite + nitrate but it was significantly increased in ASA-sensitive platelets. In addition, ASA-sensitive platelets have abolished their aggregating response to submaximal concentrations of collagen whereas platelets resistant to ASA were more sensitive to collagen activation. According, previous works have demonstrated a more sensitive response to collagen by ASA-resistant than ASA-sensitive platelets [20]. Taken together, in the resting conditions and in ASA-resistant platelets the lower content of phosphorylated NOS3 at Ser^1177^ and the reduced effect of collagen to stimulate platelet aggregation may be in accordance with highest susceptibility of the platelets to be activated. Therefore, our findings may reveal the alteration of platelet NO production as new mechanism to explain the higher susceptibility of ASA-resistant platelets to be activated which it may contribute to the increased risk to develop thrombotic-related cardiovascular events that they have been reported associated with platelet resistance to ASA [11], [16], [33].

### Study considerations and limitations

As in previous works mentioned, the main limitation of the present study may be the methodology used to classify ASA-sensitive and ASA-resistant groups. However, the PFA-100 analysis has been extensively used to determine platelet response to ASA. In this regard, several meta-analyses have demonstrated the association between ASA-resistant platelets using PFA-100 device with higher risk of cardiovascular events [16]. However, the here observed results should be only associated with PFA-100 as means of classification of the platelet response to ASA.

ACEI treatment may be a confounding factor because more patients with ASA-sensitive platelets were under this treatment. However, the effect of ACEI on the here reported results may be discarded since ACEI treatment was used as covariant in the lineal regression model.

It is evident that in the present study several points remained to be clarified. First, the purity of platelets in the PRP. In this regard, the content of platelets in PRP may be contaminated with other blood cells, particularly erythrocytes and leukocytes. In this regard, a work from Gambaryian et al reported lack of expression of NOS3 protein in human platelets suggesting that in other studies that demonstrated the presence of such NOS isoform in human platelets were as result of potential contamination by leukocytes and erythrocytes [34]. However, other authors have also suggested that contamination of platelets preparations by other cells is unlikely to account for platelet NOS activity since in most of the cases NO was synthesized by the platelet samples in response to physiological platelet agonists and neither erythrocytes nor leukocytes respond to these agonists. Furthermore, different groups have demonstrated that human erythrocytes contain NOS3 but non catalytic activity to produce NO [35], [36]. Moreover, the results of the flow cytometric analysis demonstrated that the presence of erythrocytes and leukocytes was very low and similar to those previously reported by us and by other authors [37], [38]. Although, it could not discard at all the influence of other blood cells than platelets in the here reported findings, the number of leukocytes and erythrocytes identified in the PRP is probably very small to be attributed to them. As mentioned, another point to be clarified is the fact that under resting conditions the release of NO by platelets seems to be similar between ASA-sensitive and ASA-resistant platelets whereas NOS3 phosphorylation at Ser^1177^ was significantly highest in ASA-sensitive platelets. As above mentioned, NOS3 activity is dynamically regulated and not only take part NOS3 phosphorylation but also many others factors including cell localization of NOS3 in invaginations of the platelet plasmalemma, termed caveolae, where NOS3 interacts with caveolins attenuating NOS3 activation [39]. As speculation, and mainly based on the results observed during collagen stimulation, it is possible that in ASA-resistant platelets the cycle of NOS3 activation may be disrupted in more than one step, which is more evident during platelet activation, and the slight increase in NOS3 Ser^1177^ phosphorylation in the ASA-resistant platelets after collagen stimulation may be reflex of it.

In summary, ASA-sensitive platelets showed higher content of phosphorylated NOS3 protein at Ser^1177^ than ASA-resistant platelets. This difference was markedly enhanced during platelet stimulation with collagen. In our knowledge, these findings provide for the first time an association between the platelet response to ASA and the platelet content of phosphorylated NOS3 Ser^1177^.
